# Born Toon Soon: Care before and between pregnancy to prevent preterm births: from evidence to action

**DOI:** 10.1186/1742-4755-10-S1-S3

**Published:** 2013-11-15

**Authors:** Sohni V Dean, Elizabeth Mary Mason, Christopher P Howson, Zohra S Lassi, Ayesha M Imam, Zulfiqar A Bhutta

**Affiliations:** 1 Albert Einstein Medical Center, Philadelphia, USA; 2 World Health Organization; 3 March of Dimes Foundation, USA; 4 Aga Khan University, Karachi 74800, Pakistan; 5 The Hospital for Sick Children, Toronto, Canada

## Abstract

Providing care to adolescent girls and women before and between pregnancies improves their own health and wellbeing, as well as pregnancy and newborn outcomes, and can also reduce the rates of preterm birth. This paper has reviewed the evidence based interventions and services for preventing preterm births; reported the findings from research priority exercise; and prescribed actions for taking this call further. Certain factors in the preconception period have been shown to increase the risk for prematurity and, therefore, preconception care services for all women of reproductive age should address these risk factors through preventing adolescent pregnancy, preventing unintended pregnancies, promoting optimal birth spacing, optimizing pre-pregnancy weight and nutritional status (including a folic acid containing multivitamin supplement, and ensuring that all adolescent girls have received complete vaccination. Preconception care must also address risk factors that may be applicable to only some women. These include screening for and management of chronic diseases, especially diabetes; sexually-transmitted infections; tobacco and smoke exposure; mental health disorders, notably depression; and intimate partner violence. The approach to research in preconception care to prevent preterm births should include a cycle of development and delivery research that evaluates how best to scale up coverage of existing, evidence-based interventions, epidemiologic research that assesses the impact of implementing these interventions, and discovery science that better elucidates the complex causal pathway of preterm birth and helps to develop new screening and intervention tools. In addition to research, policy and financial investment is crucial to increasing opportunities to implement preconception care, and rates of prematurity should be included as a tracking indicator in global and national maternal child health assessments.

**Declaration:**

This article is part of a supplement jointly funded by Save the Children's Saving Newborn Lives programme through a grant from The Bill & Melinda Gates Foundation and March of Dimes Foundation and published in collaboration with the World Health Organization (WHO). The original article was published in PDF format in the WHO Report "Born Too Soon: the global action report on preterm birth (ISBN 978 92 4 150343 30). The article has been reformatted for journal publication and has undergone peer review according to *Reproductive Health*'s standard process for supplements and may feature some variations in content when compared to the original report. This co-publication makes the article available to the community in a full-text format.

## The importance of preconception health and care before pregnancy

**Figure 1:**
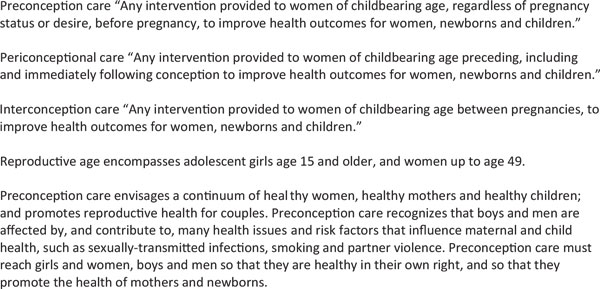
**Scope and definitions of preconception care**. Source: Bhutta et al., 2011a.

Preconception care has, until recently, been a weak link in the continuum of care. Providing care to women and couples before and between pregnancies (interconception care) improves the chances of mothers and babies being healthy, and awareness is growing. Preconception care may be defined as "any intervention provided to women and couples of childbearing age, regardless of pregnancy status or desire, before pregnancy, to improve health outcomes for women, newborns and children"[1], or "a set of interventions that aim to identify and modify biomedical, behavioral and social risks to a woman's health or pregnancy outcome through prevention and management" [2]. An expanded scope and definitions for preconception care are provided in Figure 1.

Preconception care emphasizes outcomes for maternal and child health; however, it is vital to recognize that all girls and boys have the right to grow and develop in good health, just as all women and men have the right to be healthy -- physically, psychologically and socially. Extending the RMNCH continuum to the preconception period improves the health and wellbeing of mothers, newborns and children as well as the health and wellbeing of girls and women, and boys and men, in their own right.

**Figure 2:**
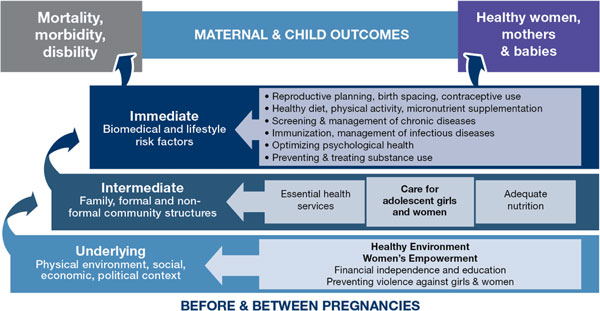
**Conceptual framework for preconception care**. Source: Born Too Soon: The global action report on preterm birth, 2012.

As shown in Figure 2, the conceptual framework for preconception care encompasses broader initiatives such as women's education and empowerment, and more targeted health interventions such as vaccination and micronutrient supplementation. Preconception care allows the time necessary for behavioral interventions to take effect. In various countries, it has been provided in schools, primary health care facilities or community centers, and has involved husbands, health care providers, youth leaders and community volunteers in achieving healthier outcomes for mothers and babies.

Many women, however, are unaware of how their health before conception may influence their risk of having an adverse outcome of pregnancy. As shown by the RMNCH continuum of care [3], health education and other programs delivered to all women during adolescence, before conception and between pregnancies can improve women's own health during pregnancy as well as that of their babies [4-6]. The imperative for preconception health is even greater given that 41% of all women report that their pregnancies were unplanned [7]. Thus, waiting to provide needed health interventions until a woman and her partner decide to have a child will be too late in 4 out of 10 pregnancies.

**Figure 3:**
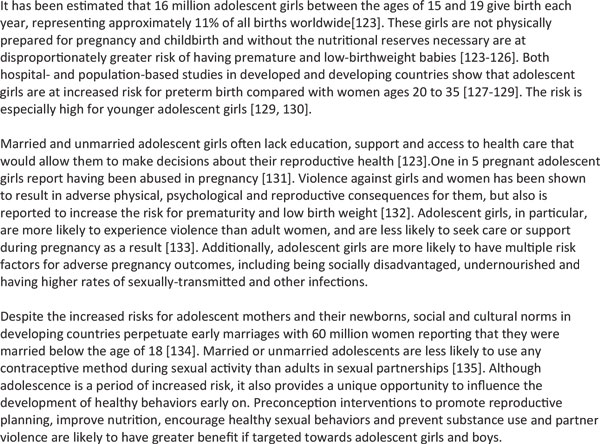
**Importance of preconception care for adolescent girls**.

Preconception care simultaneously promotes reproductive planning and interventions to reduce risk, allowing women to enter pregnancy in the best possible health and to have the best possible chance of giving birth to a healthy newborn. Outreach and awareness must begin in adolescence if it is to truly improve the health of women and newborns and reduce the rates of prematurity and low birthweight (Figure 3). The contextual and individual risks that increase the likelihood of preterm births and other adverse pregnancy outcomes are present from the time a girl reaches adolescence, and they continue during and between pregnancies.

The objectives of this paper are to review the evidence based interventions and services for preventing preterm births; report the findings from research priority exercise to list and rank important research options in preconception care; and prescribe actions for taking this agenda further.

## Priority packages and evidenced-based interventions

There is growing evidence that reducing risks in the preconception period improves the health of the pregnant woman and also contributes to the prevention of preterm birth. Table 1 presents risk factors associated with an increased risk of preterm birth. These estimates were derived from a detailed review of the evidence base on preconception risk factors for all adverse outcomes of pregnancy and landmark reviews on the causes of preterm birth [1,8-10].

**Table 1:** Risk factors associated with an increased risk of preterm birth and the effectiveness of intervention arrayed according to the strength of evidence

		How great is the risk?
**Pregnancy in adolescence**	+	Increased prevalence of anemia, pregnancy-induced hypertension, low birthweight, prematurity, intra-uterine growth retardation and neonatal mortality
**Birth spacing**	+	
Short intervals		PTb: OR 1.45, LBW: OR 1.65
Long intervals		PTb: OR 1.21, LBW: OR 1.37
**Pre-pregnancy weight status**	+	
Underweight		PTb: OR 1.32, LBW: OR 1.64
Overweight & obesity		PTb: OR 1.07
		Maternal overweight is a risk factor for many pregnancy complications including hypertensive disorders, gestational diabetes, postpartum hemorrhage, stillbirth, congenital disorders Both underweight and overweight women have a higher chance for requiring obstetric intervention at delivery
**Micronutrient deficiencies**	+/-	
Folic acid		Folic acid deficiency is definitively linked to neural tube defects (NTDs) in newborns
Iron		Anemia increases the risk for maternal mortality, low birthweight, preterm birth and child mortality
**Chronic diseases**	+	
Diabetes mellitus		Babies born to women with diabetes before conception have a much higher risk of stillbirths, perinatal mortality, congenital disorders, as well as spontaneous pregnancy loss, preterm labor, hypertensive disorders, and delivery by cesarean birth.
Hypertension		
Anemia		A study shows that anemia before conception increases the risk of low birthweight (OR 6.5)
**Poor mental health **(especially depression) **and Intimate partner violence**	++	Increased risk for preterm birth, low birthweight and depression during pregnancy and the postpartum period IPV-PTb OR 1.37, LBW OR: 1.17 Also increased risk for spontaneous pregnancy loss, stillbirth, gynecological problems including sexually-transmitted infections, depression
**Infectious diseases**	++	
STIs - syphilis		Infectious diseases increase the risk for spontaneous pregnancy loss, stillbirths and congenital infection
HIV/AIDS		
Rubella		
**Tobacco use**	++	A single study shows risk PTb OR: 2.2 Smoking increases the risk for spontaneous pregnancy loss, placental disorders, congenital malformations, sudden infant death syndrome, stillbirths and low birth weight

For magnitude of risk:

++ means strong evidence of risk and implicated in biological pathways leading to preterm birth and low birthweight

+ means moderate evidence of risk on preterm birth and low birthweight

+/- means weak evidence of risk on preterm birth and low birthweight

Acroynms used: PTb = preterm birth; OR = odds ratio; IPV = intimate partner violence

Source: Barros et al., 2010; Bhutta et al., 2011a; Goldenberg et al., 2008; Iams et al., 2008

Factors that have been shown to be strongly predictive of preterm risk, but cannot be modified, include history of previous spontaneous preterm birth, cervical procedures, including biopsies, primiparity, grand multiparity, and multiple gestations. Factors associated with socioeconomic and racial disadvantage will, hopefully, be amenable to positive transformation over the longer term, but this will require fundamental structural changes to society and a deep-seated shift in social values and norms.

Table 2 presents the priority evidence-based interventions and packages during the preconception period and before pregnancy that have potential to reduce preterm birth rates. These include interventions currently recommended by the WHO in the preconception period (e.g., family planning and prevention and treatment of STIs) [11]. Only interventions with evidence of strong or moderate effectiveness are described in the section below. Efforts are now underway to develop guidelines for preconception care and expand the package of interventions to include those listed in Table 2 -- for example, optimizing pre-pregnancy weight, screening for and treating mental health disorders and other chronic diseases like diabetes and hypertension, preventing intimate partner violence and promoting cessation of tobacco use and exposure to secondhand smoke in the home and workplace. It should be noted that because preconception care is a relatively new concept, the evidence base for risks and interventions before conception is still being strengthened. Thus, broad consensus regarding a package of evidence-based interventions for care in the preconception period has yet to be decided.

**Table 2:** Priority interventions and packages during the preconception period and before pregnancy to reduce preterm birth rates

**Preconception care services for the prevention of preterm birth for all women**
• Prevent pregnancy in adolescence
• Prevent unintended pregnancies and promote birth spacing and planned pregnancies
• Optimize pre-pregnancy weight
• Promote healthy nutrition including supplementation/fortification of essential foods with micronutrients
**Preconception care services for women with special risk factors that increase the risk for preterm birth**
• Promote vaccination of children and adolescents
• Screen for, diagnose and manage mental health disorders and prevent intimate partner violence
• Prevent and treat STIs, including HIV/AIDS
• Promote cessation of tobacco use and restrict exposure to secondhand smoke
• Screen for, diagnose and manage chronic diseases, including diabetes and hypertension

## Preconception care services for prevention of preterm birth for all women

### Prevent pregnancy in adolescence

Preconception care that begins early on and continues between pregnancies will help to ensure that women have a reproductive life plan and are able to decide when to have children, how many children they desire and methods used to prevent unintended pregnancy. In some regions, cultural norms promote early marriage, which is a factor in high rates of adolescent pregnancy. Regulations to increase the legal age at marriage and educating communities to change cultural norms that support early marriage may be ways to prevent adolescent pregnancy in those countries. In an effort to discover what interventions are most effective to prevent adolescent pregnancy, a wide variety of programs carried out in low-, middle-, and high-income countries has revealed that the most successful programs are responsive to the unique educational, social, economic, nutritional, psychological and medical needs of adolescents [12]. Particular emphasis must also be placed on ensuring universal access to primary and secondary education for girls through increasing formal and informal opportunities, because girls who complete their education are less likely to become pregnant in adolescence [13,14] While expanded sexual education programs increase adolescents' knowledge of risk, they have not been shown to change behaviors. In a combined analysis, personal development programs that incorporated skills-building and include contraceptive provision were shown to prevent 15% of first adolescent pregnancies [15,16] and programs that taught parenting skills and enabled teen mothers to complete their education decreased repeat adolescent pregnancies by 37% [1,17,18]. Across all contexts, programs demonstrated greater success if they were holistic in scope rather than solely focused on sexual education and STI/teen pregnancy prevention. It is important to note that programs with a longer duration were more effective since adolescents require time to integrate new information, practice the skills that will allow them to negotiate safe behaviors and develop confidence in themselves to broaden their life options [1,15-18].

One way to ensure that mothers and babies have good outcomes is to encourage pregnancy planning. Women who have very closely spaced pregnancies (within 6 months of a previous live birth or pregnancy) are more likely to have preterm or low-birth weight babies [19]. This may be because they have not had enough time to replenish their nutritional reserves or treat an infection or other systemic illness. Therefore, adequately spaced pregnancies have beneficial impacts on health and survival of the living child. Family planning and contraception does not prevent future pregnancy but foster mother to provide more time to herself and to her living child. The correct, consistent use of family planning methods leads to more women spacing their pregnancies 18 to 24 months apart, which is ideal [20]. Encouraging family planning and the use of contraceptive methods (hormonal and barrier methods) has other advantages including reductions in maternal and infant mortality, lower rates of unintended pregnancies, and prevention of STIs, including HIV [19,20].

Breast feeding promotion for 24 months can prevent closely spaced pregnancies, a method that continues to be underused despite strong evidence of its positive effect on maternal and newborn health. On its own, 12 months of contraception-only coverage in the preceding birth interval can reduce the mortality risk for the next newborn by 31.2%, whereas 12 months of contraceptive use overlapping with breastfeeding reduces the risk by 68.4% [20]. Programs to make effective contraception available to women and couples of reproductive age must also include counseling and follow-up to determine if the chosen method of contraception is being used correctly, and so that the method may be changed if necessary. It has been demonstrated that contraceptive counseling by trained care providers in the immediate postpartum period, or as part of comprehensive care after pregnancy loss, increases women's uptake and their partner's support for contraceptives [1]. Appropriate birth spacing after a previous live birth or pregnancy loss decreases the risk for prematurity in subsequent pregnancies [21,22].

Although contraceptive use, particularly amongst adolescents, currently falls far short of the optimal with only 56% of the demand for family planning satisfied among the Countdown to 2015 priority countries [23], the renewed interest in family planning and contraceptive commodity security (UK Govt Family Planning Summit held in July 2012, UN Commission on life-saving Commodities for Women and Children) gives an unprecedented opportunity to scale up use of contraception and allows for women and partners to plan their pregnancy. Strategies for improving coverage, especially in low-resource settings, are urgently needed and require vigorous research.

### Optimize pre-pregnancy weight

Optimizing weight before pregnancy is recommended, since weight gain or loss during pregnancy increases the risk of adverse pregnancy outcomes. Monitoring nutritional status through measurement of women's body mass index prior to pregnancy is feasible, even in low-income contexts, and should be used as a baseline to develop a regimen for healthy eating and physical activity to optimize their weight.

Women who are underweight before pregnancy (body mass index less than 18.5 kg/m^2^) are at significantly greater risk of having premature, low birth weight newborns [24]. Given that maternal undernourishment is a risk factor for being underweight, improving food security could reduce the rates of preterm birth, especially in impoverished nations. It is important, therefore, to evaluate whether local and national food programs largely targeted towards children could be replicated for adolescent girls and women.

Obesity is a problem of increasing magnitude globally with estimated 300 million women of reproductive age who are obese [25]. Overweight and obese women (body mass index greater than 25 kg/m^2^) have a higher risk for preterm births [26,27]. While existing evidence indicates that weight loss at any age is difficult to achieve and sustain, successful programs for women in their reproductive years reaffirm that women can overcome environmental pressures like easy access to low-cost, high-calorie foods and develop healthy eating habits. These programs promote dietary modification and increased physical activity through sustained daily changes, with the help of a support system and regular monitoring [28-36]. Women should be encouraged to include moderate physical activity in their daily routine to improve weight and cardiovascular status before pregnancy and reduce the likelihood of developing weight-related complications during gestation [37]. Programs should be tailored to women's weight at baseline and their lifestyle, to build motivation and increase the chances of sustaining weight loss.

### Promote healthy nutrition including supplementation/fortification of essential foods with micronutrients

Studies of the biological mechanismsleading to preterm birth indicate that more severe congenital disorders, including neural tube defects, might result in preterm delivery [38]. Consuming a multivitamin containing 400 μg of folic acid in the preconceptional period is the best way to ensure adequate micronutrient intake to help prevent neural tube and other birth defects [39]. Multi-vitamin supplementation reduces the risk of congenital malformations (e.g., neural tube, congenital heart, urinary tract and limb defects) by 42-62% and the risk of preeclampsia by 27%. Folic acid supplementation or fortification reduces the risk of neural tube defects by 53% [1]. Although folic acid is known to protect against neural tube defects, there is little evidence to show that folic acid supplementation alone reduces the risk for preterm birth [40]. In addition, providing folic acid supplementation to all women of childbearing age poses a major logistical challenge. In middle- and low-income countries, iron and folic acid supplementation reaches fewer than 30% of women [41]. Even in the United States where there are aggressive promotional campaigns, only 1 in 3 women of childbearing age takes a vitamin with folic acid daily [42].

For this reason, iron and folic acid fortification of foods for mass consumption is considered an important strategy to increase micronutrient levels in the population. A number of countries have already opted to increase population folic acid intake through inexpensive, large-scale fortification, which has proven to be moderately effective and safe [43-57]. However, legislation for mandatory fortification of food staples has still not been enacted in many countries. Further information is needed on other and multiple micronutrients in relation to preconception care especially among adolescent girls

### Promote vaccination of children and adolescents

Infections transmitted around the time of conception or during pregnancy may result in preterm birth [58]. Not only does infection, especially with rubella virus, increase the risk for prematurity, it may lead to other devastating consequences such as congenital rubella syndrome or miscarriage [39,59,60]. Many of these infections could be prevented through routine childhood vaccinations. However, the rubella vaccine can also be given at least 3 months prior to pregnancy to women who are not already immune [61]. Vaccination campaigns against rubella have been able to increase coverage for adolescent girls and women [62-66].

## Preconception care services for women with special risk factors that increase the risk for preterm birth

### Screen for, diagnose and manage mental health disorders and prevent intimate partner violence

Maternal stressors such as depression, socioeconomic hardship and intimate partner violence have been linked to preterm birth [67-71]. It has been hypothesized that physical and psychological stress acts through inflammatory pathways involving maternal cortisol to cause premature birth [72,73]. Importantly, when such risks are present before pregnancy they are likely to continue throughout pregnancy as well. Moreover, women with psychosocial stressors have a greater likelihood of engaging in risky behaviors such as smoking and alcohol use and are less likely to seek health care [74,75]. Risky sexual behaviors also put these women at greater risk for unintended pregnancies and STIs [76-78]. Interventions to improve the psychological health of women before conception have included group counseling and development of coping and economic skills. These have shown some promise in reducing risk, but so far have not demonstrated reductions in adverse birth outcomes including prematurity. Further research in this area is needed since the burden of mental health disorders -- particularly depression, anxiety and somatic disorders -- is high in women, and the safety of some medications used to manage these conditions during pregnancy is unclear. A Joint Statement by the American Psychiatric Association and American College of Obstetrics and Gynecology indicates that the higher risk of preterm birth may be related to depression itself, or the antidepressants used for treatment [79]. Behavioral therapy for couples before marriage, for men who have been violent with their partners, and for married couples in a violent relationship has shown a reduction in aggression, largely in more severe forms of violence [80-83]. Two programs that integrated interventions for domestic violence and substance use also showed some success, however, the effect generally faded with time [84,85].

### Prevent and treat STIs, including HIV/AIDS

Reducing the incidence of infectious diseases, particularly syphilis, is a high priority to lower the rates of stillbirths and preterm birth [86]. A number of interventions have been piloted in various countries to prevent and treat STIs, especially since such interventions also impact teen pregnancy, HIV/AIDS and contraceptive use. Focusing interventions on high-risk groups, including women, adolescents and intravenous drug users, can effectively reduce the transmission of STIs to the population in general and subsequently reduce preterm births and stillbirths [87,88]. Behavioral and counseling interventions may lead to a 25% rise in the practice of safe sexual behaviors and a 35% drop in the incidence of STIs [1]. Mass treatment interventions with antibiotics also have been shown to decrease the prevalence of STIs by one-fifth [89-91]. Counseling and behavioral interventions that focus on educating women are especially crucial, given that women are physically more vulnerable to contracting a STI during intercourse than men, and are less likely to have the ability to negotiate safe behaviors with their partners such as condom use [92]. Focused interventions for preventing the broad range of STIs may be helpful in preventing preterm births, though more research is needed.

### Promote cessation of tobacco use and restrict exposure to secondhand smoke

Cigarette smoking approximately doubles the threat of preterm birth [93]. Despite the risk of fetal growth restriction and preterm birth [94-96], a survey of women in low- and middle-income countries found that many pregnant women currently used tobacco or were exposed to secondhand-smoke [97]. A few studies have shown, however, that preconception counseling and the involvement of husbands or partners in smoking cessation programs can increase the number of women who quit smoking before pregnancy [98,99]. In many instances even when women themselves do not use tobacco, they are exposed to environmental tobacco smoke and indoor air pollution; interventions and regulatory measures must therefore target male partners and behavioral change on a wider level to minimize women's exposure.

### Screen for, diagnose and manage chronic diseases

In the United States alone, 12% of women of reproductive age suffer from diabetes and hypertension [100]. Although testing and treatment for women diagnosed with such medical problems prior to pregnancy are cost-effective and prevent further complications for the mother and baby, they do not necessarily lower the incidence of preterm births [10]. For example, achieving good control of diabetes through counseling, weight management, diet and insulin administration could reduce the risk of perinatal mortality and congenital disorders by approximately 70%, but does not significantly lower the rate of preterm birth among diabetic mothers [1]. At any contact with health care services, women of reproductive age should, therefore, be asked about other medical conditions and the use of medications particularly about anti-epileptic drugs. Women with epilepsy not only face the possible risk for adverse pregnancy outcomes as a result of the teratogenic effects of antiepileptic drugs upon the developing fetus but also the potential direct effects of maternal seizures on the developing fetus. Until adequate control of the medical condition is achieved, women should be educated about the possible risks to themselves and their newborn, and be encouraged to use effective contraception [101]. Multivitamin supplementation for women with chronic medical conditions is especially important because it has been shown to lower their risk for adverse pregnancy outcomes [102]. For women with other chronic conditions, such as cardiorespiratory disease, systemic lupus erythematosus, hypertension and renal disease, a cesarean birth may be indicated leading to the a baby being born prematurely; however, even in such cases, achieving optimal control of the condition before pregnancy may lead to better long-term outcomes for the mother and newborn.

## Limitations of the evidence

The growing interest in preconception care is fairly recent; thus, there are limited data specific to the period prior to and between pregnancies, particularly relating to preterm birth risks and outcomes. Risk factors and interventions that have been studied only in adolescents or only during pregnancy also may be relevant in the preconception period. For instance, exposure to indoor air pollution during pregnancy leads to 20% more stillbirths and low birth weight babies [103]. Yet many women are exposed to biomass smoke and second-hand tobacco smoke long before pregnancy is established. Similarly, interventions such as smokeless stoves or smoking cessation programs that reduce overall levels of exposure also would benefit women who later become pregnant. For many women, a positive pregnancy test is a stimulus to cease smoking, yet most women require multiple attempts to quit. Smoking cessation programs for adult men and women have been evaluated and demonstrate higher rates of women who quit before or during the first trimester [104]. Given the strong evidence of risk for preterm birth and low birth weight with tobacco use in pregnancy, it may be inferred that fewer women smoking translates to lower rates of preterm birth.

Many interventional studies in the preconception period report different health outcomes, which is also the case for studies on pregnancy and childbirth [105]. This precludes a complete assessment of the impact that an intervention could have on multiple pregnancy outcomes. For instance, research to reduce the prevalence of STIs among women may assess safe sexual behaviors or rates of transmission as outcomes; however, many studies do not indicate how many women later became pregnant or change in rates of preterm birth.

Until now, preconception care has been provided through three avenues: pre-pregnancy health visits for couples contemplating pregnancy; programs to increase awareness, screening and management for a particular risk; or participatory women's groups in the community. The diversity of contexts and risks among adolescent girls and women will require that preconception care be tailored to different settings and groups. The approaches used are a step in the right direction; but could be broadened to include earlier health care and health promotion for women and couples and address risks more holistically.

## Program opportunities to scale up

There is widespread agreement that in order to reduce maternal and childhood mortality, a continuum of care needs to be provided and that actions are needed at the community, primary care and referral care levels to deliver this continuum [4]. Packages of interventions to improve maternal and newborn health have been developed; yet, these focus largely on care during pregnancy and after birth [106]. However, it is important to realize that most of the health risk behaviors that are emphasized during pregnancy are generally the ones that are advised before pregnancy. Steering the action from preconception period will improve gearing the risk factors soonest and can lead to profound benefits for health and well-being of women and couples and improve subsequent pregnancy and child health outcomes. Tracking progress and scaling up delivery of preconception interventions has been a challenge, with preconception initiatives in individual countries delivering different services to different segments of the population (women, couples or adolescents).

In some high-income countries, such as the United States, Hungary, Australia and the Netherlands, an attempt has been made to provide preconception care to couples of reproductive age through family physicians or a special preconception clinic [98,107-111]. Evidence-based recommendations for the content of preconception care also have been published [2,112-114], and components have been incorporated into major national and international health guidelines [11,115]. In the United States, a website has been developed to support clinical education and practice in this area http://www.beforeandbeyond.org.

In some countries (India, Pakistan, Bangladesh and Nepal), women's support groups have been teaching birth preparedness to women and their partners [101,116-119]. Many large-scale trials for individual preconception interventions also have been carried out in low- and middle-income countries. While individual settings will require context-specific approaches to providing preconception care, a number of effective and culturally-acceptable interventions already exist. An example of an opportunity to build on existing programs is the integration of interconception health into home visits during the postnatal period.

The evidence base for risks and interventions before conception is still being strengthened because preconception care, as noted, is a relatively new concept. Therefore, an agreed-upon package of evidence-based interventions and opportunities for scale up in the preconception period has yet to be decided.

## Priorities for research for preconception care

There is limited evidence on the effectiveness of preconception care in reducing preterm births, which presents a major barrier for reducing the global burden of preterm birth. Since preconception care is still an emerging field across the research pipeline--from description to delivery, development and discovery--there is much to be done (Table 3). A research priority-setting exercise was conducted to list and rank important research options in preconception care, with 76 technical experts systematically and transparently scoring 381 options using the Child Health and Nutrition Research Initiative (CHNRI) method [120]. The list of research priorities (Table 4) emphasizes improvement and delivery of existing interventions to women in contexts with constrained resources, since the highest burden of maternal and child mortality and morbidity including preterm births occurs amongst women of lower socio-economic status. Experts in maternal and child health strongly suggest operational research to improve nutrition; prevent adolescent pregnancy; increase uptake of contraception; screen for chronic conditions (such as hypertension and anemia); treat infectious diseases (notably HIV/AIDS); and update immunizations during the preconception period. Experts also advocated for scaling up coverage of effective interventions through integration of preconception interventions with other platforms and programs; task-shifting to community health workers; utilizing cell phones and information technologies; improving the supply chain for preconception care commodities; and maximizing uptake by adolescents.

**Table 3:** Research priorities for preterm outcomes related to preconception

**Description**
• Maintain and expand global databases on the prevalence of preconception risk factors and incidence of preterm birth
• Develop indicators to evaluate progress in scaling up coverage of preconception care
• Evaluate impact of preconception care programs on rates of preterm birth and other adverse pregnancy outcomes
**Discovery**
• Basic science research on preconception risk factors for preterm birth
**Development**
• Develop and test screening tool to assess risk of preterm birth based on risk factors in the preconception period
• Develop ways to increase demand for and access to preconception interventions
**Delivery**
• Define and test preconception care guidelines and intervention packages
• Explore means to integrate effective preconception interventions into broader programs and initiatives
• Adapt effective interventions to maximize uptake by adolescents
• Improve health systems -- including infrastructure, management, distribution of goods and training of providers -- to deliver preconception care

**Table 4:** The top research priorities based on the expert CHNRI process for preconception care in low- and middle-income countries to reduce maternal and child mortality and morbidity

1.	How can preconception nutrition interventions, such as diet diversity, micronutrient supplementation/fortification and achieving optimal BMI, be integrated into broader nutrition and/or health programs and delivered in a cost-effective manner?
2.	What are the public health approaches to regulate and reduce exposures to environmental tobacco smoke?
3.	How can effective interventions to prevent adolescent pregnancy and repeat adolescent pregnancy be delivered at scale?
4.	What are the public health approaches to regulate and reduce environmental exposures to smoke stoves?
5.	What approaches work to increase the use of effective contraception, especially long-acting methods, particularly in the postnatal and post-abortion time periods?
6.	What are effective, affordable and feasible means to screen for hypertension affecting girls and women before conception?
7.	What are the most effective strategies to scale up the prevention/detection/treatment of malaria and helminthiasis to reduce anemia in women of reproductive age?
8.	What effective strategies can be developed to modify individuals' behavior to reduce their environmental exposures to smoke stoves?
9.	What effective, affordable strategies could be developed to provide effective STI/HIV identification and management, including early antiretroviral therapy, as part of preconception care, and how could these be adapted to maximize uptake by adolescents?
10.	How can task-shifting to community health workers to screen for chronic conditions among women during the preconception period and take appropriate action (such as referring to specialist, counseling, refer to support groups) be effectively enabled?
11.	How can the effect and cost of different delivery strategies for an immunization package for girls, including rubella and tetanus and others as appropriate, be best developed and evaluated?
12.	How can the supply chain for commodities for effective preconception services (e.g., nutrition, contraception, medications for chronic and infectious diseases) be integrated with other logistical systems so that it is more reliable and effective?

For some important risk factors that have been identified, epidemiologic data are lacking. National, regional and global databases are needed that track adolescent girls and women exposed to a particular risk (for example pre-pregnancy underweight, anemia or infection) and rates of preterm birth in high-risk versus healthy mothers. Additionally, monitoring systems must be in place to evaluate the effect of introducing or scaling up interventions on the incidence of preterm birth and other pregnancy outcomes. Epidemiologic measurement is critical to establish goals, track progress and compare intervention strategies. Replicating these interventions in larger studies of adolescent girls and women before first pregnancy, or between pregnancies, is needed to assess the relative benefit that may be obtained through preconception care across different populations.

There is a need for discovery research to further elucidate the etiology of preterm birth and identify ways to screen women. There is also a great need for innovative interventions and new ways to implement existing interventions, especially ways to assess and reduce exposure to risk factors that are not directly amenable to medical intervention, such as environmental pollution.

The development of national and international guidelines specific to preconception care would increase the visibility of the issue for health care providers and the population in general. While there is need for a defined and tested preconception care package, that can be adapted to various settings and models of service delivery at scale, much is still undiscovered, both in terms of what interventions work to reduce risks such as pre-pregnancy underweight and obesity or mental health problems and how to integrate effective preconception interventions into broader programs and initiatives across the continuum of RMNCH.

The interventions with proven benefit and national data, such as family planning, require further operational research including how to maximize provision of preconception care in the healthcare setting and community, and how to promote uptake by adolescents and women particularly those who are at high-risk for a preterm birth. The feasibility of scaling up preconception interventions will need to be assessed, including improvements to infrastructure, supply chain and health management systems also may increase coverage of preconception services.

Piloted interventions to improve the health of adolescent girls and women, which can lead to prevention of preterm birth, are often not categorized as preconception care; thus, they present a missed opportunity for linking to preterm birth research. Continued research will be necessary to identify tailored interventions for women from different strata and with different risk profiles within the same communities. There also is need to develop simple, accessible and user-friendly ways to provide individualized preconception care to women in contexts where resources are lacking or where health systems are weaker.

Addressing contextually-relevant ways to increase demand for and access to preconception care services is especially necessary in developing countries. While many countries have implemented behavior change strategies to increase awareness on birth preparedness and women's empowerment, more strategies for assessing benefit particularly for preterm birth are needed, especially culturally appropriate ways to involve adolescent boys, men and communities.

Even with current tools used to diagnose disease such as hypertension, the development of simpler, cost-effective diagnostic tests will enable efficient point-of-care testing with timely results and minimize the need for multiple visits. Likewise, affordable, easy-to-administer preventive and treatment options that are woman-friendly are in demand, such as oral insulin or better female-controlled contraceptive methods. With the knowledge gaps for preconception care, there is room for testing innovative technology and for implementation research.

## Prescription for action

Although preconception care is now recognized as a way to better the health of women and couples and improve pregnancy and newborn outcomes, a package of essential preconception interventions has not yet been agreed upon, nor is there global consensus on how to incorporate preconception care into the overall maternal, newborn and child health strategy. A meeting was organized by the World Health Organization in early 2012 to meet these objectives, and develop an action plan for moving the agenda for preconception care forward [121]. The meeting brought together leading researchers in the field, individuals with programmatic implementation experience of preconception care, and a number of organizations working to improve maternal and child health worldwide who were interested in developing an action plan for preconception care. There was a strong sense of the importance of preconception care, and a common understanding of preconception care as part of a continuum of care to improve the health of adolescents, women, couples of reproductive age, mothers, newborns and children. It was agreed that there is a need to distinguish between proximal preconception care that would occur one to two years before conception and distal preconception care that would extend even earlier, since the target populations and interventions for each may differ. Target population groups should include all women and men of reproductive age who may or may not be currently contemplating pregnancy with special efforts to reach vulnerable groups such as adolescent girls, those who are socioeconomically marginalized, and couples with previous adverse reproductive and pregnancy outcomes. It is important to specifically target adolescent and young women because health habits initiated during that period of age have profound impact on future health and their future pregnancy outcomes. Risky behaviors such as use of alcohol, tobacco and illicit drugs are significant challenges to their health, while, risky sexual behavior put them at high risk for unintended pregnancy and HIV/STIs. It was emphasized that preconception care is needed in low- and middle-income countries which have the highest burden of maternal and child mortality and morbidity, but it also relevant and important for women in high-income countries who are socioeconomically deprived. Further, all participants wanted to agree on an essential package of preconception interventions, with the possibility for regions and countries to select additional preconception care services and delivery strategies based on contextual factors. It would therefore be important that preconception care programs are documented, evaluated and disseminated so that others can learn from such experiences in adapting preconception care services to their setting.

Effective preconception interventions that decrease maternal and child mortality and morbidity should be delivered using appropriate methods that include health education, vaccination, nutritional supplementation and food fortification, contraceptive information and services, screening and management of medical and social risk factors. There are certain risk factors which lead to some behavioral effects and complex outcomes. To illustrate, risky sexual behavior and cases of intimate partner violence are higher among those who consume alcohol and are also involved in substance abuse. Disentangling these factors and effects may be difficult. Hence ameliorating these linked risk factors and behaviors can prevent a host of factors for poor pregnancy outcomes. In the health care setting, an essential package of interventions and a checklist of risk factors to be screened for might be a feasible starting point. In some countries, mandatory screening for hereditary diseases before marriage drastically reduced rates of thalassemia, and whether preconception care could be delivered through a similar means has not been explored [122]. Packaging these interventions further ensures translation of knowledge into action. The packages feature opportunities for delivery and highlight the implementation of intervention packages via existing health care and public health programs. A package of existing antenatal program, for example, if includes preconception care can be an effective strategy in low-income countries and would capture a wide audience. School health and reproductive health programs, on the other hand, can guide adolescent as to how to make responsible decisions concerning their sexual lives, as well as how to practice safe sex, and how to prevent unwanted pregnancies.

Provision of preconception care must also be extended beyond those who are traditionally involved in women's health, by incorporating the concept into training for current and future health care providers. For some risks, such as chronic diseases, until diagnosis and treatment can become more affordable, policymakers and donor organizations must work in conjunction to make screening and care widely available. Increasing coverage of postpartum care would also help to improve women's health in future pregnancies, for example, through the integration of preconception care in postnatal home visits. Men are critical and equal partners in family planning and reproductive health practices and their involvement in preconception care can bring positive outcomes. They play a major role in supporting women with which they can not only foster healthy practices but can also discourage risky behaviors such as smoking and intake of alcohol in their partners. Furthermore involving males can exert positive impact on their own behaviors and practices and hence influence the family at large.

National preconception care services will depend on local resources, health systems and existing public health strategies or maternal and child programs. The WHO will recommend a package of essential preconception interventions that are evidence-based, universally relevant (for example family planning and optimization of maternal nutritional status) and that can be delivered even in resource-poor settings. A list of action points that were posited and additional points especially related to reducing the rates of preterm birth, are shown in Table 5.

**Table 5:** Actions before and between pregnancy to reduce the risk of preterm birth

**Invest and plan**
• Assess situational need for preconception care services and opportunities in local health system to deliver.
• Use every opportunity to reach girls and women and couples with preconception messages, beginning in school and extending to healthcare settings and community events. Preconception health must also involve boys and men, to improve their health; and to engage them in ensuring better outcomes for women and girls.
• Develop consensus around the use of a term and a definition for preconception care grounded in a conceptual framework.
• Publish the existing evidence base, and identify gaps in the evidence base.
• Raise the profile of preconception care and engage key stakeholders to support action and research in this area (including through advocacy documents, scientific publications, participation in meetings of professional organizations; engaging experts and organizations in fields outside of maternal and child health).
• Prepare guidelines on preconception care.
• Develop a list of tools to support policy development, implementation, monitoring and capacity-building in preconception care.
**Implement**
*Seize opportunities through existing programs (including non-health programs) to:*
• Educate women and couples of reproductive age to have a reproductive plan that includes age at first pregnancy, method to prevent unintended pregnancy, and number of children they wish to have.
• Scale up personal development programs and skills-building to negotiate safe sexual behavior in adolescence. Adapt preconception interventions to maximize uptake by adolescents.
• Implement universal coverage of childhood and booster vaccinations for infectious diseases known to cause adverse pregnancy outcomes.
• Screen for and treat infectious diseases, particularly sexually transmitted infections.
• Promote healthy nutrition and exercise to prevent both underweight and obesity in girls and women.
• Promote food security for communities and households. Expand nutrition programs to include adolescent girls and women. Particularly for underweight women, provide protein calorie supplementation and micronutrients. A cost-effective way to ensure adequate levels of micronutrient consumption would be to enact large-scale fortification of staple foods.
• Implement public health policy to reduce the number of men and women of reproductive age who use tobacco.
• Implement strategies for community development and poverty reduction, since living environments and socioeconomic constructs have a significant impact on health.
• Ensure universal access to education to empower girls and women with the basic knowledge and skills they need to make decisions for themselves, such as when to access care.
*Scale up*
• Promote effective contraception for women/couples to space pregnancies 18 to 24 months apart.
• Screen for chronic conditions, especially diabetes, and institute counseling and management as early as possible to improve neonatal outcomes.
**Inform and improve program coverage and quality**
• Develop indicators for baseline surveillance and to monitor progress in preconception care.
• Include preterm birth among tracking indicators.
• Develop a common analytical framework to evaluate existing preconception care programs and document their processes and outcomes to inform and inspire others.
• Develop national and global indicators to track progress in delivery of preconception care.
**Innovate and undertake implementation research**
• Invest in research and link to action.
• Identify opportunities to incorporate in-service and pre-service training on preconception care within existing capacity-building efforts, including through distance education.
• Stimulate and support country-level action.
• Carry out demonstration projects to strengthen the evidence base for the value and feasibility of preconception care.
We all share in the responsibility of making sure that all women before and between pregnancies receive the care they need for healthy pregnancies and birth outcomes.

Also of importance will be the encouragement of dialogue and collaboration with other sectors -- for example, education food and agriculture and telecommunications and media -- which are already engaged with young people of preconception age to promote greater demand for preconception care, and to reach girls, women and couples beyond the health care system. In some cases, integrated programs have already been shown to be feasible and effective, such as youth development programs, contraceptive provision in school for adolescents and incorporating maternal health into child vaccination days. It is also essential to develop a way to involve men, community leaders, volunteers and families in support for and provision of preconception care.

## Conclusion

Until recently, the provision of care to women and couples before and between pregnancies to improve maternal and newborn health has not had sufficient priority on the RMNCH continuum of care. As with research, care must focus increasingly "upstream" from birth if the true potential for prevention of preterm birth is to be realized. Effective preconception care involves a broad variety of partners, including men, health care providers, youth leaders and community volunteers; and delivery sites such as schools, primary health care facilities and community centers. Outreach and awareness must begin in adolescence if it is to truly improve the health of women and newborns and reduce the rates of prematurity. If tackled, however, with vigorous and evidence-based interventions, preconception care offers the earliest opportunity to reduce risk, allowing women to enter pregnancy in the best possible health and to have the greatest chance of giving birth to a healthy baby.

## Competing interests

The authors declare that they have no competing interests.

## Supplementary Material

Additional file 1 **In line with the journal's open peer review policy, copies of the reviewer reports are included as **additional file 1. Click here for file
