# Supplementary material for: Born Toon Soon: Care before and between pregnancy to prevent preterm births: from evidence to action
Source: Reprod Health. 2013 Nov 15;10(Suppl 1):S3. doi: 10.1186/1742-4755-10-S1-S3 (PMC3828587; doi:10.1186/1742-4755-10-S1-S3)
Supplement: Additional file 1 — In line with the journal's open peer review policy, copies of the reviewer reports are included as additional file 1. [file 1742-4755-10-S1-S3-S1.doc]

**Referee’s comments to the authors– this sheet WILL be seen by the author(s) and published with the article**

| Title | **Care before and between pregnancy** |
| --- | --- |
| Author(s) | Sohni Dean, Zulfiqar Bhutta, Elizabeth Mary Mason, Christopher Howson, Zohra Lassi, Ayesha Imam |
| Referee’s name | Vijay Kumar |

**When assessing the work, please consider the following points, where applicable:**

1. Is the question posed by the authors new and well defined?
2. Are the methods appropriate and well described, and are sufficient details provided to replicate the work?
3. Are the data sound and well controlled?
4. Does the manuscript adhere to the relevant standards for reporting and data deposition?
5. Are the discussion and conclusions well balanced and adequately supported by the data?
6. Do the title and abstract accurately convey what has been found?
7. Is the writing acceptable?

Please make your report as constructive and detailed as possible in your comments so that authors have the opportunity to overcome any serious deficiencies that you find and please also divide your comments into the following categories:

- Major Compulsory Revisions (which the author must respond to before a decision on publication can be reached)
- Minor Essential Revisions (such as missing labels on figures, or the wrong use of a term, which the author can be trusted to correct)
- Discretionary Revisions (which are recommendations for improvement but which the author can choose to ignore)

Where possible please supply references to substantiate your comments.

When referring to the manuscript please provide specific page and paragraph citations where appropriate.

**Reviewer comments**

The article is very well written. This is a very relevant contribution to a missing piece in the continuum of care that would impact the health of women and men during reproductive period and beyond.. The methods are appropriate and well described. There are sufficient details and implications for referral. These relate to policy implementation and research. The data is reviewed in details and interpreted in a balanced manner. The reporting meets the standards of writing. The discussion and conclusions are balanced and adequate The tile and the abstract are accurate and the writing is quite acceptable,

**The revisions suggested by the reviewer require minor essential revisions and discretionary revisions to improve the article and make it stronger to convince the national and international policy makers funders and decision makers.**

Throughout the article, the terms preconception care and care between the pregnancies should be used consistently since most of the risk factors have adverse consequences on pregnancy outcomes. The term pre conception care does not by itself include care in between two pregnancies.

The targeted groups and the age when the intervention packages should be considered in the policy for implementation are important. A broader approach should include adolescents and young people and a discussion on the possible impact of targeting interventions for a broader age group in contrast to a focused approach that is implemented over a short period in the continuum of care This has been mentioned but some elaboration and emphasis indicating the pros and cons would be very useful to the readers and would strengthen the contribution.

The scope of adverse outcome of pregnancy should be broadened to include (a) still births (b) prematurity and intrauterine growth retardation (c) birth defects and (d) neonatal and maternal mortality. The information on the above adverse outcomes in a large number of countries continues to be weak. The above outcomes are all amenable to the intervention packages proposed in the article. The inclusion of the above outcomes in the national data bases would lead to more convincing advocacy for pre conception care and care in between pregnancies. In the article there seems to be emphasis on pre term births but the other components do not receive as much attention. While revising the article, a balance should be reached.

The continuum of care emphasis is highly relevant but it is perhaps important to state that the risk factors not only are relevant to pre conception phase and in between two pregnancies but also throughout pregnancy. In the current antenatal care programmes attention would be required to continue to control the risk factors mentioned in the article (those that are relevant to the stage)

Although male involvement has been mentioned it has not been adequately emphasized .This can become a major bottle neck if not included in the policy and programmes. The relevance of male involvement can be stressed in the context of the use of tobacco, intimate partner violence, risky sexual behavior and male support especially during the pre conception and in between pregnancy phase of life.

The health and survival of the living child is adversely affected when another pregnancy occurs as a result of poor child spacing The use of effective contraception to space two pregnancies will not only affect the future pregnancy outcome but also help the mother to provide adequate care to the living child (as well as remain focused on her own health) including exclusive breast feeding for a period upto two years of age.

Food fortification is clearly the way forward but rolling it out has a gestational period. In the interim period the policy should support the program for folic acid and multi vitamins with greater vigour and intensity to impact on adverse outcomes.of pregnancy

The authors should consider integrating the approach to the control of risk factors because the risk factors included tend to cluster and the effects are likely to cumulate. There is a relationship between use of alcohol, substance use, intimate violence, risky sexual behaviours and psycho social stressors. The clustering has commonality in influencing not only pregnancy outcomes but also the higher risk for STI and HIV/AIDS.

Amongst the psycho social risks the authors may kindly include epilepsy since it can influence pregnancy outcomes on its own as well as a result of the anti epileptic drugs also

Malaria and helminthaisis are important risk factors as identified in the table but this is not mentioned anywhere in the text. These problems deserve a serious consideration in countries where these conditions are a serious public health problem.

.

**Referee’s comments to the authors– this sheet WILL be seen by the author(s) and published with the article**

| Title | **Care before and between pregnancy** |
| --- | --- |
| Author(s) | Sohni Dean, Zulfiqar Bhutta, Elizabeth Mary Mason, Christopher Howson, Zohra Lassi, Ayesha Imam |
| Referee’s name | Jennifer Requejo |

**When assessing the work, please consider the following points, where applicable:**

1. Is the question posed by the authors new and well defined?
2. Are the methods appropriate and well described, and are sufficient details provided to replicate the work?
3. Are the data sound and well controlled?
4. Does the manuscript adhere to the relevant standards for reporting and data deposition?
5. Are the discussion and conclusions well balanced and adequately supported by the data?
6. Do the title and abstract accurately convey what has been found?
7. Is the writing acceptable?

Please make your report as constructive and detailed as possible in your comments so that authors have the opportunity to overcome any serious deficiencies that you find and please also divide your comments into the following categories:

- Major Compulsory Revisions (which the author must respond to before a decision on publication can be reached)
- Minor Essential Revisions (such as missing labels on figures, or the wrong use of a term, which the author can be trusted to correct)
- Discretionary Revisions (which are recommendations for improvement but which the author can choose to ignore)

Where possible please supply references to substantiate your comments.

When referring to the manuscript please provide specific page and paragraph citations where appropriate.

**General comments:**

This paper provides a comprehensive overview of knowledge to date on the importance of preconception care for preventing preterm delivery and improving health outcomes for women and their babies.

The paper summarizes the evidence, and presents some ideas on what interventions should be included in a package of preconception care services and some thoughts on delivery strategies. It is an important and timely topic given the rising rates of preterm birth, and the need to focus on the preconception period given that preventive and treatment interventions provided during pregnancy may already be too late to reduce preterm birth rates and most effectively improve maternal and newborn health.

A couple of overarching thoughts to strengthen the paper:

1. The title needs to be a little bit more informative on the content of the article
2. Structure of the article – it currently reads more like a summary chapter in a book or report. It might be useful to include information in the introductory paragraphs on what the article is about, how the evidence was collected and analyzed, and the basic structure.
3. The concept of preconception care and consistency with the definition. Box 1 provides a definition limiting preconception care to delivery of services to women of reproductive age, but most o

**Minor essential revisions:**

**Discretionary revisions:**

*(continue on the next sheet)*

*Continued*: of the remaining text in the box is about the health of women, girls, men and boys. Can the authors reconcile this? Can the authors speak to criticisms with the label of ‘preconception care’ as narrowly constructing the importance of services for girl’s and women’s health before and in-between pregnancies as mainly to ensure healthy pregnancies rather than for their own health? (i.e., take a look at the bulletin special issue on women’s health beyond reproduction)

4. Preconception care and its relationship to the RMNCH continuum of care. I’m not sure it is technically correct to argue that the construction of the CoC has, until recently, not included preconception care services. The RMNCH CoC has always included family planning, and an emphasis on the pre-pregnancy, pregnancy, childbirth, postnatal and up to 2 year time period. This focus on the pre-pregnancy and up to 2 year window corresponds with evidence on the most critical window of time to end preventable maternal and child deaths. Other evidence, such as from the nutrition community, suggests that there should be a focus on the first 1,000 days.

It seems what the authors are suggesting is either 1) that there needs to be a continuation of care throughout childhood, adolescence, and adulthood and that this is what will lead to improved chances of positive birth outcomes for mom and baby, or 2) that there seems to have been a gap in thinking on what services should be provided during the ‘pre-pregnancy’ phase of the CoC and all the dimensions this phase encompasses (i.e., adolescent health, women in-between pregnancies, etc.). Can the authors clarify? The first notion of care throughout the lifecycle has the drawback of making it a challenge to narrow down what the scope of preconception services would constitute, or if what is really being advocated for is universal access to health care (as well as to education and other services that would lead to greater empowerment and opportunities for women).

Part of this is related to the presentation of the information – the authors provide a lot of very rich info in each section on the evidence about how specific interventions or interventions for specific populations might affect preterm birth rates. But, each section mainly delves into why these interventions should be provided regardless of impact on prematurity because they are evidence based interventions for reducing maternal and/or newborn mortality/morbidity. The downside to this approach is that it then becomes unclear if the paper is about the benefits of preconception care more generally, or about the link between preconception care and prematurity. This, I think, is related to a point I made earlier about the article not including a summary on what it is about and what approach was used to review available evidence.

5. Service packages for preconception care. It also seems that the authors could consider thinking a little further on how to present the info on possible packages of preconception care services. Based on the info in the chapter, it seems that these packages would necessarily be different for different population groups, and the integration of preconception care services into existing platforms would also be different depending upon the target group. The authors do a great job of providing a lot of good information in table 2 on priority interventions, but it is not clear who is responsible for the provision of these services, what level of the health care system they are being delivered at, or how they might be integrated into existing services. Table 4 shows the list of research priorities (not specific to preterm birth, but to explore how preconception care in LMICs can reduce MNC mortality), and these questions, when viewed together, actually do help make clear the different levels (pointing out where research is needed in the policy arena, in the health sector and in non-health sectors (i.e., agriculture, education), etc).

For example, points of contact for adolescent girls and boys could be school health programs, and clinics where they may be taken for an HPV vaccine (which is now being thought of as a new contact point for reaching adolescents). Preconception care services for women in-between pregnancies could be provided as part of the set of services provided during PNC, family planning, routine exams, although annual exams is probably not something women in low-resource settings typically do.

6. Minor point: There is a paragraph on page 2 about the programs that have been found to be most effective for adolescent health, but the information is so vague and all encompassing. Can the authors give a little bit more detail on the elements of the most effective programs?

## Comments from the Editor:

For all the articles, these should be seen as independent papers that can be read without the need to read the other papers in the series.
